# Automatic Spontaneous Speech Analysis for the Detection of Cognitive Functional Decline in Older Adults: Multilanguage Cross-Sectional Study

**DOI:** 10.2196/50537

**Published:** 2024-04-29

**Authors:** Emilia Ambrosini, Chiara Giangregorio, Eugenio Lomurno, Sara Moccia, Marios Milis, Christos Loizou, Domenico Azzolino, Matteo Cesari, Manuel Cid Gala, Carmen Galán de Isla, Jonathan Gomez-Raja, Nunzio Alberto Borghese, Matteo Matteucci, Simona Ferrante

**Affiliations:** 1 Department of Electronics, Information and Bioengineering Politecnico di Milano Milano Italy; 2 BioRobotics Institute and Department of Excellence in Robotics and AI Scuola Superiore Sant’Anna Pisa Italy; 3 SignalGeneriX Ltd Limassol Cyprus; 4 Department of Electrical Engineering, Computer Engineering and Informatics Cyprus University of Technology Limassol Cyprus; 5 Geriatric Unit Fondazione Istituto di Ricovero e Cura a Carattere Scientifico Ca’ Granda Ospedale Maggiore Policlinico Milano Italy; 6 Ageing and Health Unit Department of Maternal, Newborn, Child, Adolescent Health and Ageing World Health Organization Geneva Switzerland; 7 Consejería de Sanidad y Servicios Sociales Junta de Extremadura Merida Spain; 8 Department of Computer Science University of Milan Milano Italy; 9 Laboratory of E-Health Technologies and Artificial Intelligence Research in Neurology Joint Research Platform Fondazione Istituto di Ricovero e Cura a Carattere Scientifico Istituto Neurologico Carlo Besta Milano Italy

**Keywords:** cognitive decline, speech processing, machine learning, multilanguage, Mini-Mental Status Examination

## Abstract

**Background:**

The rise in life expectancy is associated with an increase in long-term and gradual cognitive decline. Treatment effectiveness is enhanced at the early stage of the disease. Therefore, there is a need to find low-cost and ecological solutions for mass screening of community-dwelling older adults.

**Objective:**

This work aims to exploit automatic analysis of free speech to identify signs of cognitive function decline.

**Methods:**

A sample of 266 participants older than 65 years were recruited in Italy and Spain and were divided into 3 groups according to their Mini-Mental Status Examination (MMSE) scores. People were asked to tell a story and describe a picture, and voice recordings were used to extract high-level features on different time scales automatically. Based on these features, machine learning algorithms were trained to solve binary and multiclass classification problems by using both mono- and cross-lingual approaches. The algorithms were enriched using Shapley Additive Explanations for model explainability.

**Results:**

In the Italian data set, healthy participants (MMSE score≥27) were automatically discriminated from participants with mildly impaired cognitive function (20≤MMSE score≤26) and from those with moderate to severe impairment of cognitive function (11≤MMSE score≤19) with accuracy of 80% and 86%, respectively. Slightly lower performance was achieved in the Spanish and multilanguage data sets.

**Conclusions:**

This work proposes a transparent and unobtrusive assessment method, which might be included in a mobile app for large-scale monitoring of cognitive functionality in older adults. Voice is confirmed to be an important biomarker of cognitive decline due to its noninvasive and easily accessible nature.

## Introduction

According to “The 2021 Ageing Report by the European Commission,” life expectancy has shown a continuous trend over the past years [1]. As life expectancy increases, so does the number of people with dementia worldwide. Dementia is a neurodegenerative disease, which entails a long-term and gradual decrease in cognitive functionality, resulting in the reduction of patients’ autonomy and well-being, as well as worsening of the quality of life of their caregivers. The management of the increased number of older adults at risk of developing severe cognitive decline is a big challenge for health care systems, with the annual global cost expected to rise to US $2 trillion by 2030 [2]. These pathologies start silently up to 20 years before clear cognitive symptoms. However, there is increasing evidence that pharmaceutical interventions may be most effective at milder stages of dementia [3]. Thus, it is fundamental to find strategies that may anticipate the diagnosis [4-6]. Current diagnostic procedures require a thorough examination by medical specialists. The most employed tool for the first screening of cognitive function is the Mini-Mental Status Examination (MMSE). It is based on 30 questions that address short and long-term memory, attention span, concentration, language, and communication skills, as well as the ability to plan and understand instructions [7]. A score of 26 or higher is usually classified as normal. If the score is below 25, the result highlights a possible cognitive impairment, which may be classified as mild (21≤MMSE score≤26) or moderate to severe (MMSE score≤20). Although this test has high sensitivity and specificity (87% and 82%, respectively) [8] and can be quickly administered, its employment is restricted within primary care facilities. Thus, faster, noninvasive, and automatic methods are needed to provide digital biomarkers for large-scale monitoring of cognitive functions in real-life scenarios [9].

In recent years, voice has been one of the most studied digital biomarkers since it allows cheap, noninvasive, ecological, rapid, and remote assessment of several aspects of a patient’s health status, such as the functionality of the respiratory system, cognitive decline, emotions, and heart dysfunctions [7,10-12]. Speech and language capacity is a well-established early indicator of cognitive deficits [13,14]. In the early phase of dementia, participants show alterations in the rhythm, resulting in a higher number of pauses, probably due to word-finding problems (ie, anomia and semantic paraphasia), worsening of verbal fluency [15-17], low speech rates, and decrease in the length of voiced segments [18-20]. Several studies have addressed the possibility of identifying signs of cognitive decline from voice recordings. Martínez-Sánchez and colleagues [21] analyzed the temporal parameters of reading fluency to discriminate between Spanish-speaking asymptomatic participants and those with Alzheimer disease (AD), and they were able to differentiate between patients with AD and healthy controls with an accuracy of 80% based on the speech rate. However, using a reading task introduces the possibility that participants’ fluency is affected by other factors such as educational level or visual impairment. Konig et al [22] demonstrated that it is possible to differentiate between dementia and mild cognitive impairment (MCI) in English-speaking participants based on voice features extracted from different tasks, for example, verbal fluency, picture description, counting down, and free speech, with a classification accuracy of 86%. Toth et al [23] showed that acoustic parameters such as speech rate, hesitation ratio, number of pauses, and articulation rate yield good results in discriminating between Hungarian-speaking participants with MCI and healthy controls. They analyzed a movie recall task and achieved an *F*_1_-score of 78.8%. Calzà et al [2] were able to discriminate between Italian-speaking healthy controls and participants with MCI by using random forest and support vector machine (SVM) with an *F*_1_-score of 75% by employing natural language processing. Finally, Bertini et al [24] achieved high performance (accuracy of 93% and *F*_1_-score of 88.5%) based on acoustic features extracted from spontaneous speech from a corpus of English-speaking participants, that is, Pitt Corpus, by using deep learning techniques. Nevertheless, natural language processing and deep learning require the analysis of raw data, thus having access to the recordings’ information content and endangering the participants’ privacy. Most previous works [2,21-24] aimed to distinguish participants with a proper diagnosis of AD or MCI from healthy participants. However, as far as we know, there are no studies investigating whether machine-learning algorithms based on voice features can identify early signs of functional cognitive decline detected by a decrease in the MMSE score.

In a previous study of our group [25], voice features automatically extracted from recordings of episodic storytelling could discriminate between Italian-speaking participants with normal cognitive functions (MMSE score≥27) and participants with mild cognitive decline (20≤MMSE score≤26) with an accuracy of 73%. Starting from this preliminary study, our study exploits acoustic features automatically extracted from spontaneous speech and machine learning techniques to support the early identification of cognitive function decline, meant as a reduction of the MMSE score. The main novelties involve the extension of a number of features, reduction of the computational time for feature extraction, and the multilanguage approach since both Spanish- and Italian-speaking participants were considered.

## Methods

### Participants and Data Collection

A sample of older adults were recruited in Italy (Lombardy region) and Spain (Extremadura region). In Italy, participants were recruited based on direct contact with the Geriatric Unit of the Foundation Scientific Institute for Research, Hospitalization and Healthcare (IRCCS) Ca’ Granda Ospedale Maggiore Policlinico (day hospital, ambulatory, and gymnasium). In Spain, people were recruited based on direct contact with professionals working in health care belonging to the Extremadura Health Ecosystem.

The essential requirement for participation was a good knowledge, at least oral, of the language of the country where the audios were recorded. Exclusion criteria were nonnative-speaking participants, clinically unstable participants, terminal illness (life expectancy <6 months), severe hearing or visual deficits, aphasia, and a score on the 30-item Geriatric Depression Scale >9. After providing informed consent to participate in the study, participants were met individually and they underwent the MMSE performed by health care professionals (geriatrician in Italy and neuropsychologist in Spain). Afterward, they were asked to tell 3 stories about their life for 2 minutes each without interruptions (positive, negative, and episodic) and to provide a 2-minute description of the “Cookie-Theft picture” of the Boston Diagnostic Aphasia Examination [26]. For each task, voice signals were recorded in separate .WAV files (16 kHz) by using an ad-hoc toolbox developed in MATLAB (MathWorks), through an external USB microphone. Participants were divided into 3 groups based on the MMSE score:

1. Group 1: MMSE score≥27, that is, healthy participants
2. Group 2: 20≤MMSE score≤26, that is, participants with mild impairment of cognitive function
3. Group 3: 11≤MMSE score≤19, that is, participants with a moderate to severe impairment of cognitive function

The choice of the MMSE score for separation among the groups was employed since the aim was to detect the earliest symptoms of cognitive decline in the prediagnostic phase.

### Ethics Approval

This study was approved by the ethics committee of Fondazione IRCCS Ca’ Granda Ospedale Maggiore Policlinico in Italy (ref: 1272018, approval date: March 15, 2018) and by the Comité Ético de Investigaciòn Clìnica de Badajoz in Spain (approval date: April 11, 2018).

### Feature Extraction and Statistical Analysis

Data preprocessing and features extraction were performed employing an automatic algorithm implemented in MATLAB [25]. A positive speech polarity was imposed, and voice recordings were standardized. Afterward, the acoustic features described in Table 1 were extracted [20,21,23,27,28]. The features were grouped into 4 macrocategories according to their information content: voice periodicity, shimmer-related, syllabic, and spectral features. Feature extraction was repeated 3 times for voice segments lasting 5 seconds, 10 seconds, and 15 seconds to assess whether different time lengths can capture specific patterns. For each voice segment length, voice features extracted from the 4 audio recordings were substituted by their mean and standard deviation or their median and interquartile range, based on data set distribution, assessed by the Anderson-Darling normality test. Thus, each participant was represented by a single entry in the final data set, and 138 acoustic features (23 features × 3 segments length × 2 statistics) were computed for each entry. A 1-way analysis of variance for independent samples was applied to compare the 3 groups in terms of age. Due to their categorical nature, the Kruskal-Wallis test was applied to compare years of education and MMSE scores among groups. A Pearson chi-squared test was instead used for gender. Finally, generalized linear mixed models were defined in SPSS Statistics (version 28; IBM Corp) to evaluate whether acoustic features were significantly different among groups. Specifically, the mean (or median) values of the 23 acoustic features extracted from the 15-second segments were considered as the target for each model following a gamma regression distribution with a log link to the linear model. If significant differences were found, post hoc analysis with Bonferroni correction was also performed.

**Table 1:** Overview of the extracted features.

Domain, feature description		Feature code
**Voice periodicity**		
	Unvoiced percentage, that is, percentage of aperiodic parts in the audio segment	F1
	Duration of voiced and unvoiced segments, that is, mean, median, 15th and 85th percentiles of the parts of the signal with (voiced) and without (unvoiced) periodic nature	F2-F9
	Percentage of voice breaks computed on the number of distances between consecutive pulses longer than 1.25 divided by the pitch floor (70 Hz) [27]	F10
**Shimmer**		
	Shimmer, that is, random cycle-to-cycle temporal changes of the amplitude of the vocal fold vibration [28]	F11
**Syllabic and pauses features**		
	Speech rate, that is, number of syllables per second [21]	F12
	Percentage of phonation time, that is, the intrasyllabic and intersyllabic nuclei time <250 ms divided by the total speech time [20,21]	F13
	Articulation rate, that is, the number of syllables divided by the phonation time without pause [20,21]	F14
	Mean duration of intersyllabic pauses >250 ms [21]	F15
	Mean duration of syllables [20,21]	F16
	Number and mean duration of pauses of the audio segment [23]	F17-F18
**Spectral features**		
	Mean (SD) of pitch	F19-F20
	Standard deviation of third formant (F3-SD)	F21
	Speech temporal regularity, that is, temporal structure of the audio segment	F22
	Centroid, that is, location of the center of mass of the spectral signal	F23

### Feature Selection and Classification

Machine learning algorithms were trained to solve multiclass and binary classification problems (group 1 vs group 2 and group 1 vs group 3) starting from the extracted voice features, which were preliminary normalized.

#### Classifiers

SVM [29], logistic regression (LR), and CatBoost classifier (CAT) [30] were used. SVM is robust to noise in training data, since SVM decisions are only determined by the support vectors, while CAT represents the state of the art of boosting algorithms based on decision trees, and it has been proven to be very effective with small data sets with a high number of features. LR was investigated due to its simplicity and low computational cost. To achieve robust estimations despite the relatively small number of samples, the performance of each classifier was evaluated using stratified nested 10-fold cross-validation, which leads to the construction of an ensemble model via soft voting starting from each fold, obtaining a macromodel composed of 10 models trained on different subsets of data [31]. The classifier was selected according to the accuracy obtained in validation. Finally, a Kruskal-Wallis test was performed to determine whether there was a statistically significant difference between different classifiers in terms of accuracy.

#### Parameter Setting

Hyperparameter tuning was performed to limit overfitting with the nonlinear classifier. The following parameters were tuned for CAT through a randomized search method: bagging temperature, tree depth, l2 leaf regularization, and random strength. SVM was employed with a linear kernel and default parameters, and LR was also considered with default parameters. All the experiments were implemented using scikit-learn Python libraries, Catboost library, and Shapley Additive Explanation (SHAP).

#### Feature Selection

Due to the high dimensionality of the features set, the selection of the most informing features was performed through SHAP [32]. For each fold, starting from the entire set of features, the training was performed iteratively by computing the accuracy and the feature importance via SHAP for that specific iteration. At the end of each iteration, the 2 least significant features were removed until the minimum number of 6 features was reached. Therefore, the best model, that is, the one that achieved the best accuracy, was selected for each fold of the outer loop, and the model parameters were tuned for the identified set of features. As a result, 10 models trained on 10 different folds, each characterized by a different set of parameters and exploiting a different set of features, were obtained. The algorithm related to a single fold of the outer loop is summarized in Textbox 1. Finally, the ranking of the most informing features was implemented by summing up the unweighted mean of the Shapley values obtained at the end of the training of each fold for each feature.

**Algorithm of feature elimination with Shapley Additive Explanations.**

1: Train algorithm with whole set of features

2: Calculate model performance

3: Calculate feature importance with Shapley Additive Explanations

4: *for* feature in range (0, total features-6) *do*

- Remove the k=2 least significant features
- Train the model with the remaining features
- Evaluate machine learning performance based on the scoring function
- Calculate new features ranking with Shapley Additive Explanations explainer

5: *end for*

6: Best set is the one with the highest scoring function

## Results

### Characteristics of the Participants

Table 2 shows the characteristics of the recruited participants. A total of 266 participants were recruited: 133 Italian-speaking and 133 Spanish-speaking older adults. In the Italian data set, most participants in all groups were females. In contrast, in the Spanish data set, participants were balanced for gender in group 1 and unbalanced in favor of females in the other 2 groups. Overall, significant differences in terms of age (P=.03 and P=.001 for the Italian and Spanish data sets, respectively), MMSE scores (*P*<.001 for both data sets), and years of education (only for the Italian data set, *P*<.001) were found among the 3 groups, with people with severe impairment of the cognitive function being characterized by an older age in both data sets and by fewer years of education in the Italian data set.

**Table 2:** Characteristics of the participants.

		Group 1^a^	Group 2^b^	Group 3^c^	*P* value	*P* value group 1 vs group 2	*P* value group 2 vs group 3	*P* value group 1 vs group 3
**Italian data set**								
	Participants, n	45	44	44	N/A^d^	N/A	N/A	N/A
	Age (years), mean (SD)	76.5 (4.9)	82.8 (4.6)	84.9 (5.7)	.03	.22	>.99	.02
	Gender (female/male)	39/6	33/11	37/7	.40	N/A	N/A	N/A
	MMSE^e^ (0-30), median (IQR)	30 (1)	24 (3)	16 (5)	<.001	<.001	<.001	<.001
	Years of education, median (IQR)	13 (3)	8 (8)	5 (5)	<.001	<.001	.36	<.001
**Spanish data set**								
	Participants, n	43	45	45	N/A	N/A	N/A	N/A
	Age (years), mean (SD)	79.9 (7.5)	82.4 (6.9)	85.6 (6.6)	.001	.05	.27	.001
	Gender (female/male)	21/22	36/9	27/18	.09	N/A	N/A	N/A
	MMSE (0-30), median (IQR)	28 (2)	23 (3)	17 (2)	<.001	<.001	<.001	<.001
	Years of education, median (IQR)	6 (5)	5 (4)	7 (4)	.25	N/A	N/A	N/A

^a^Group 1: Mini-Mental Status Examination score≥27.

^b^Group 2: 20≤Mini-Mental Status Examination score≤26.

^c^Group 3: 11≤Mini-Mental Status Examination score≤19.

^d^N/A: not applicable.

^e^MMSE: Mini-Mental Status Examination.

### Acoustic Feature Characteristics

Table 3 reports the results of the statistical analysis comparing acoustic features for the Italian and Spanish data sets. Voice periodicity features, particularly those related to unvoiced segments, were found to be significantly different among groups (P<.001 for mean, median, and 85th percentile of duration of unvoiced segments). Indeed, from group 1 up to group 3, a significant increase (P<.001 for mean and 85th percentile and P=.004 for median) in the unvoiced duration was found. Significant differences were found also for some syllabic features such as duration of pauses and syllables, which significantly increased with the decrease in the MMSE score, as expected from literature [20]. The results of the statistical analysis comparing acoustic features for the Italian and Spanish data sets separately are reported in Multimedia Appendix 1 (Tables S1 and S2).

**Table 3:** Acoustic feature characteristics and significance between the 3 groups for the Italian and Spanish data sets.

Domain, features			Group 1^a^ (n=88)	Group 2^b^ (n=88)	Group 3^c^ (n=89)	*P* value	Group 1 vs group 2	Group 2 vs group 3	Group 1 vs group 3
**Voice periodicity, mean (SD)**									
	Unvoiced (%)		32.7 (10.4)	38.2 (13.5)	44.7 (11.8)	<.001^d^	.004^d^	.003^d^	.001^d^
	**Duration of voiced segments (s)**								
		Mean	1.08 (0.36)	1.03 (0.4)	0.98 (0.79)	.24	N/A^e^	N/A	N/A
		Median	0.88 (0.32)	0.82 (0.35)	0.8 (0.75)	.33	N/A	N/A	N/A
		15th percentile	0.26 (0.1)	0.24 (0.1)	0.22 (0.11)	.003^d^	.24	.29	.002^d^
		85th percentile	2.03 (0.71)	1.93 (0.76)	1.77 (1.07)	.08	N/A	N/A	N/A
	**Duration of unvoiced segments (s)**								
		Mean	0.5 (0.14)	0.6 (0.2)	0.71 (0.23)	<.001^d^	<.001^d^	<.001^d^	<.001^d^
		Median	0.37 (0.12)	0.43 (0.13)	0.49 (0.17)	<.001^d^	.001^d^	<.001^d^	.04^d^
		15th percentile	0.15 (0.02)	0.16 (0.03)	0.17 (0.03)	.08	N/A	N/A	N/A
		85th percentile	0.91 (0.27)	1.14 (0.46)	1.42 (0.56)	<.001^d^	<.001^d^	<.001^d^	<.001^d^
	Voice breaks (%)		34.22 (10)	39.89 (13)	47.17 (11)	<.001^d^	.001^d^	.001^d^	<.001^d^
**Shimmer, mean (SD)**									
	Shimmer		5 (0.56)	5.19 (0.67)	5.05 (0.75)	.17	N/A	N/A	N/A
**Syllabic and pauses features, mean (SD)**									
	Speech rate (syl/s)		3.92 (0.59)	3.52 (0.63)	3.78 (6.76)	.15	N/A	N/A	N/A
	Phonation (%)		70 (8)	64 (9)	64 (71)	.06	N/A	N/A	N/A
	Articulation rate (syl/s)		5.61 (0.44)	5.54 (0.5)	5.41 (0.46)	.02^d^	.89	.22	.01^d^
	Mean intersyllabic duration (s)		0.14 (0.01)	0.15 (0.02)	0.16 (0.02)	<.001^d^	.001^d^	.003^d^	<.001^d^
	Mean syllabic duration (s)		0.74 (0.17)	0.91 (0.29)	1.14 (0.37)	<.001^d^	<.001^d^	<.001^d^	<.001^d^
	Number of pauses		0.62 (0.19)	0.79 (0.31)	1.01 (0.48)	<.001^d^	<.001^d^	<.001^a^	<.001^d^
	Mean duration of pauses (s)		5.09 (1.26)	5.1 (1.42)	5.32 (1.23)	.51	N/A	N/A	N/A
**Spectral features, mean (SD)**									
	**Pitch**								
		Mean	162 (25)	166 (26)	158 (25)	.11	N/A	N/A	N/A
		SD	68 (12)	72 (12)	79 (15)	<.001^d^	.10	.004^d^	<.001^d^
	F3-SD^f^		466 (46)	490(46)	484.92 (46)	.001^d^	.002^d^	>.99	.02^d^
	Speech temporal regularity		1749.5 (66)	1716.4 (67)	1687.8 (85)	<.001^d^	.01^d^	.03^d^	<.001^d^
	Centroid		807.8 (154)	776.5 (165)	755.9 (193)	.12	N/A	N/A	N/A

^a^Group 1: Mini-Mental Status Examination score≥27.

^b^Group 2: 20≤Mini-Mental Status Examination score≤26.

^c^Group 3: 11≤Mini-Mental Status Examination score≤19.

^d^Significant at P<.05.

^e^N/A: not applicable.

^f^F3-SD: standard deviation of third formant.

### Multiclass Classification

**Figure 1:**
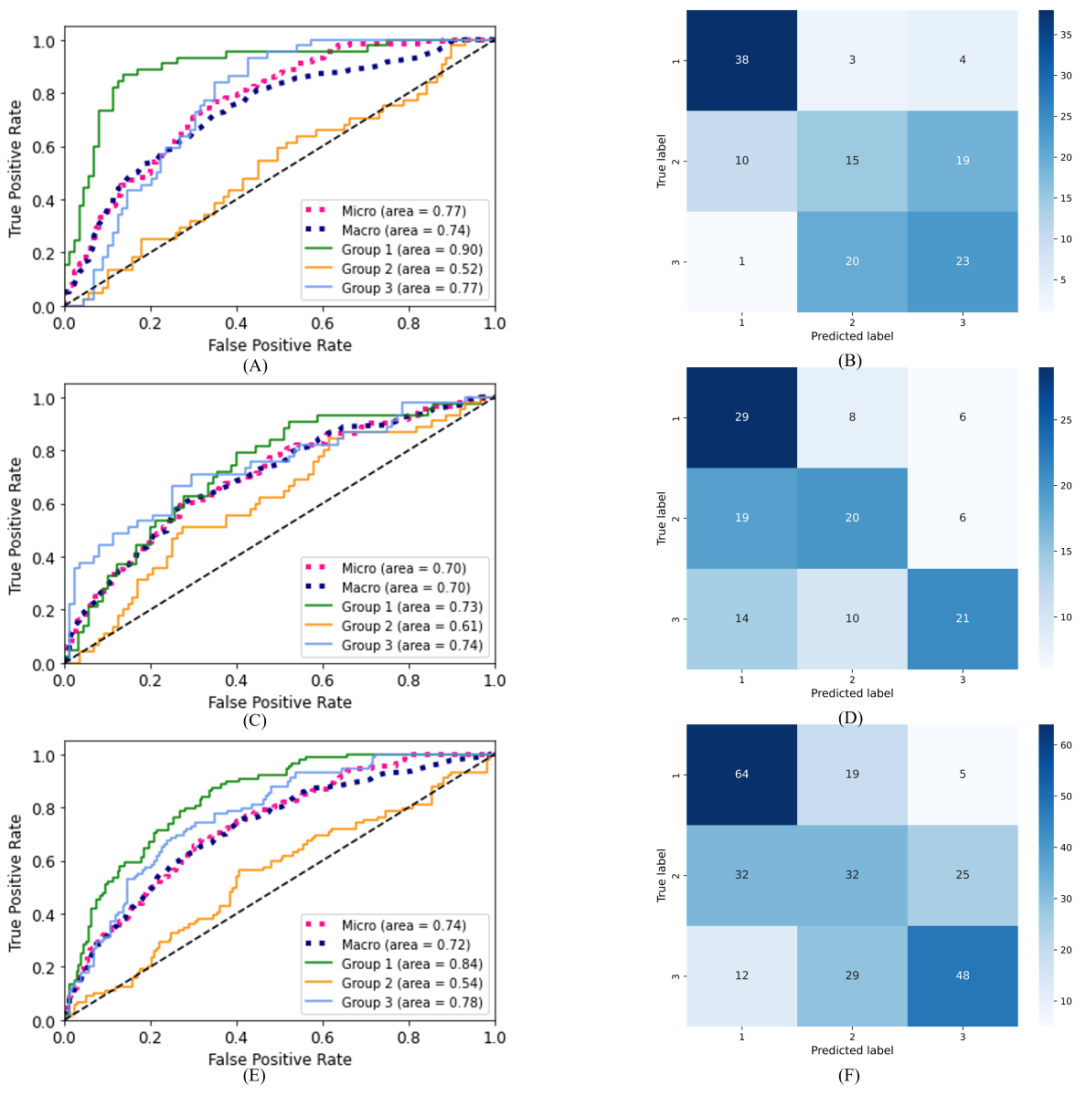
Receiver operating characteristic curves and confusion matrices obtained with support vector machine for multiclass classification of the (A,B) Italian, (C,D) Spanish, and (E,F) Italian&Spanish data sets, respectively. (A,C,E): The dotted pink line corresponds to the microaveraged receiver operating characteristic curve, while the dotted blue curve corresponds to the macroaveraged one. (B,E,F): Labels 1, 2, and 3 on the x and y axes correspond to the group number.

**Figure 2:**
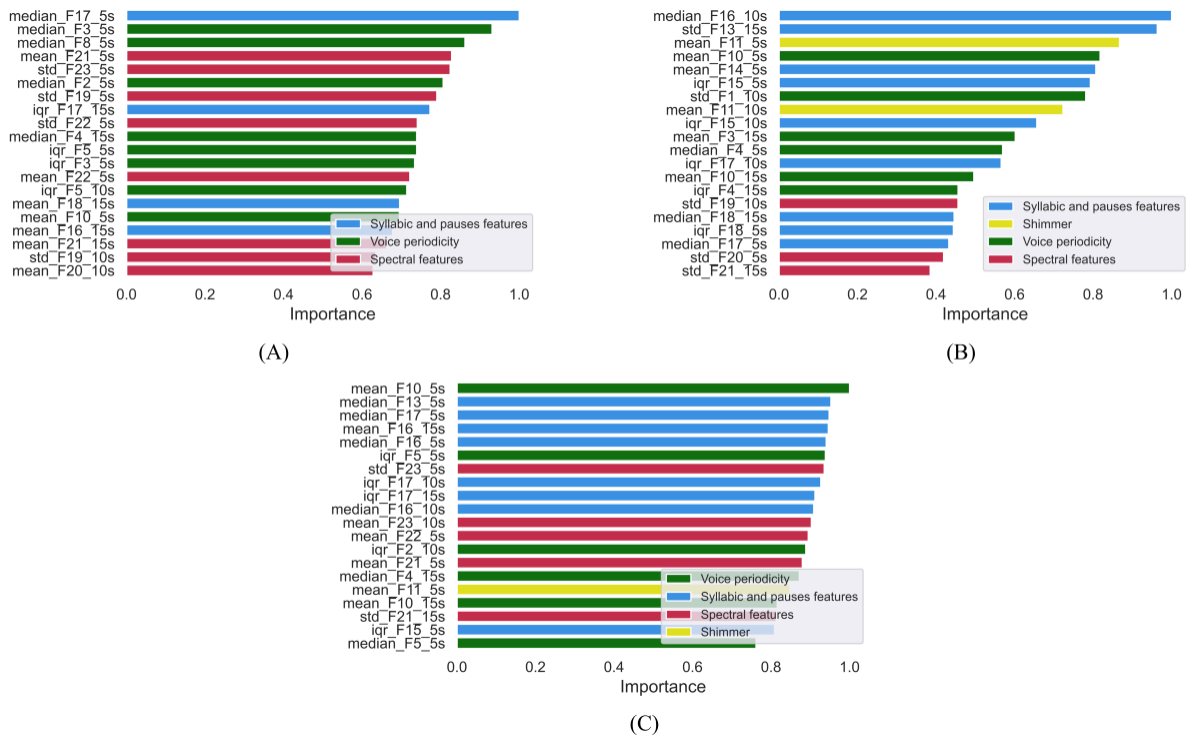
Feature ranking for (A) Italian, (B) Spanish, and (C) Italian&Spanish data sets. Rank is represented from top to bottom from the most contributing to the least important feature.

Table 4 reports the results of the multiclass classification in terms of accuracy for 3 data sets: only Italian, only Spanish, and combination of Italian and Spanish participants. Overall, CAT achieved the best scores on the validation sets for the 3 data sets, but its performance considerably worsened when applied to the test sets. From the Kruskal-Wallis test, it can be seen that CAT achieved significantly better performance than LR for all data sets (P=.005, P=.02, and P=.03 for the Italian, Spanish, and Italian&Spanish data sets, respectively). A significant difference was also highlighted between SVM and CAT for the multilanguage data set (P=.01) and between SVM and LR for the Spanish data set (P=.003). Since there was no substantial difference in the accuracy between SVM and CAT, SVM was selected for its simplicity and further metrics, that is, receiver operating characteristic (ROC) curves, confusion matrices, and feature rankings are also reported (Figures 1-2). Overall, ROC curves (Figures 1A, 1C, and 1E) show a better trend for groups 1 and 3, whereas for group 2, the curve almost overlaps the bisector. The macro and micro averages of the areas under the curves achieved a fair score. The confusion matrices in Figures 1B, 1D, and 1F confirm this trend, with group 2 being the most misclassified in all 3 cases. For the Italian data set (Figure 1B), the model mainly misclassifies the participants from group 2 with those belonging to group 3, whereas for the Spanish data set (Figure 1D), participants from group 2 were mainly misclassified with participants from group 1.

The feature rankings obtained from SHAP (Figure 2) show the contribution of the most important features, ranked from the most to the least informing. It can be seen that the most important features changed depending on the considered language. For the Italian data set (Figure 2A), the spectral features (in purple), and those related to voice periodicity (in green) were among the most important features, whereas within the Spanish data set (Figure 2B), features related to syllables and pauses (in blue) and shimmer (in yellow) became more important. For the Italian&Spanish data set, the resulting ranking was a combination of the previous two, as displayed in Figure 2C.

**Table 4:** Classification accuracies on the validation and test sets for the multiclass classification among the 3 groups (healthy, mild, and severe symptoms) for the 3 data sets.

Data set		CAT^a^	SVM^b^	LR^c^	*P* value	CAT vs SVM	CAT vs LR	SVM vs LR
**Italian, mean (SD)**								
	Validation	0.67 (0.03)	0.64 (0.02)	0.63 (0.03)	.006	.46	.005	.12
	Test	0.54 (0.08)	0.57 (0.16)	0.59 (0.13)	N/A^d^	N/A	N/A	N/A
**Spanish, mean (SD)**								
	Validation	0.63 (0.02)	0.64 (0.02)	0.60 (0.02)	.002	.88	.02	.003
	Test	0.49 (0.09)	0.53 (0.11)	0.51 (0.15)	N/A	N/A	N/A	N/A
**Italian&Spanish, mean (SD)**								
	Validation	0.61 (0.01)	0.58 (0.02)	0.58 (0.02)	.008	.01	.03	.92
	Test	0.53 (0.06)	0.54 (0.08)	0.52 (0.09)	N/A	N/A	N/A	N/A

^a^CAT: CatBoost classifier.

^b^SVM: support vector machine.

^c^LR: logistic regression.

^d^N/A: not applicable.

### Binary Classification

Tables 5 and 6 report the performance achieved for the binary classification, respectively, to distinguish group 1 (MMSE≥27) and group 2 (20≤MMSE≤26) and group 1 and group 3 (11≤MMSE≤19). SVM achieved the best scores on the validation sets compared to CAT and LR for the Italian&Spanish data sets in the discrimination between group 1 and group 2. However, the discrimination between group 1 and group 3 achieved a substantial equivalence among the 3 algorithms. As expected, better performance was obtained in the discrimination between healthy participants and those with severe impairment. As for the multiclass scenario, the accuracy of the test sets worsened in all data sets, with the Spanish data set experiencing the largest decrease.

**Figure 3:**
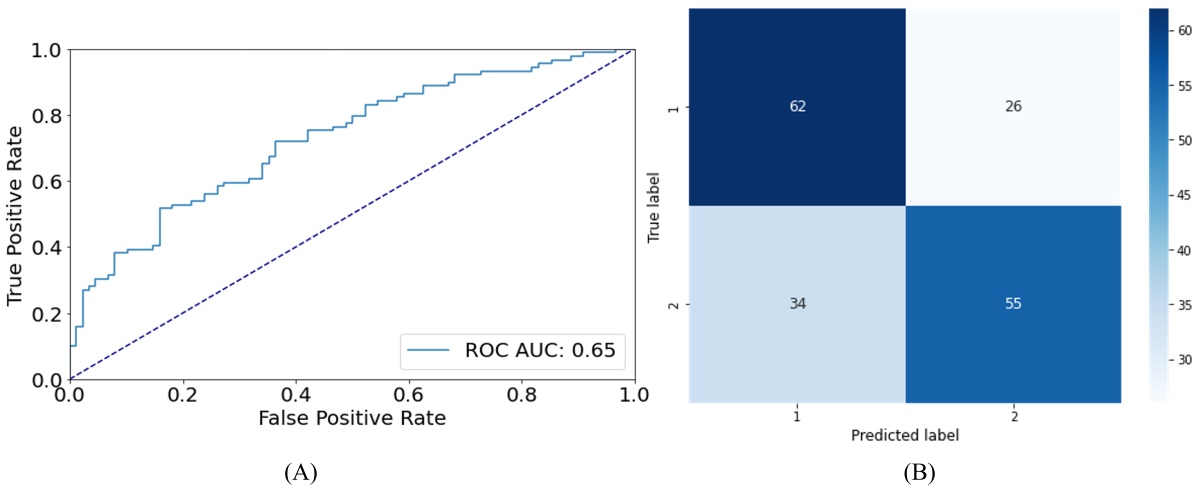
(A) Receiver operating characteristic curve and (B) confusion matrix for the binary classification of group 1 (Mini-Mental Status Examination score≥27) and group 2 (20≤Mini-Mental Status Examination score≤26) of the Italian&Spanish data set. AUC: area under the curve; ROC: receiver operating characteristic.

**Figure 4:**
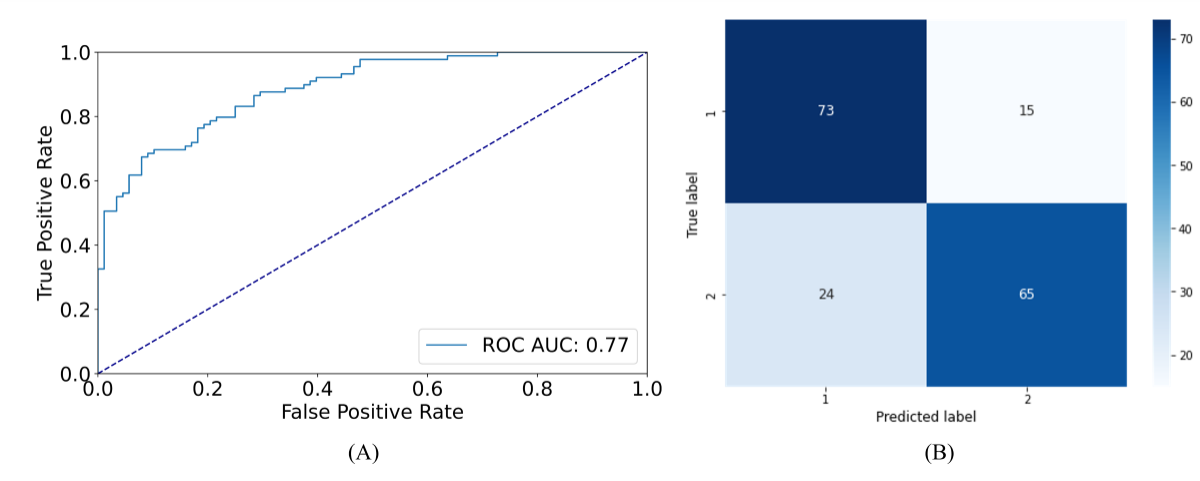
(A) Receiver operating characteristic curve and (B) confusion matrix for the binary classification between group 1 (Mini-Mental Status Examination score≥27) and group 3 (11≤Mini-Mental Status Examination score≤19) of the Italian&Spanish data set. AUC: area under the curve; ROC: receiver operating characteristic.

**Figure 5:**
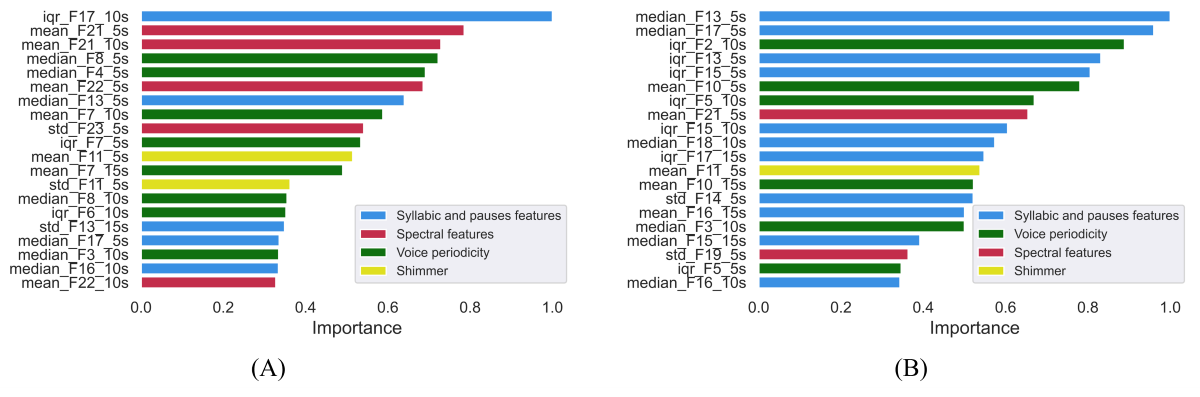
Feature ranking for the binary classifications of the Italian&Spanish data set. (A) Group 1 versus group 2; (B) group 1 versus group 3.

ROC curves, confusion matrices, and feature rankings for the Italian&Spanish data set were shown for SVM, which achieved the best performance, at least in distinguishing group 1 from group 2. Regarding ROC curves, the results were poor for the classification between healthy participants and those with mild impairment, with an area under the curve score of 0.65 (Figure 3A), as it can be noticed also by the confusion matrix in Figure 3B. Fair results were obtained for the ROC curve concerning the distinction between healthy participants and participants with impairment, with an area under the curve score of 0.77 (Figure 4A). Moreover, the confusion matrix (Figure 4B) shows a smaller number of misclassified participants. Feature rankings showed that the most informing features were mainly spectral features and features related to voice periodicity for the classification between healthy participants and participants with mild impairment (Figure 5A). In contrast, features related to syllables and pauses (in blue) were more important for classifying between healthy and older adults with severe impairment (Figure 5B). ROC curves, confusion matrices, and feature rankings related to binary classifications of the Italian&Spanish data sets are reported in Multimedia Appendix 1.

**Table 5:** Classification accuracies on the validation and test sets for the binary classification of group 1 (Mini-Mental State Examination score≥27) versus group 2 (20≤Mini-Mental State Examination score≤26).

Data set		CAT^a^	SVM^b^	LR^c^	*P* value	CAT vs SVM	CAT vs LR	SVM vs LR
**Italian, mean (SD)**								
	Validation	0.80 (0.02)	0.84 (0.02)	0.79 (0.02)	<.001	.007	.91	.02
	Test	0.71 (0.14)	0.80 (0.14)	0.76 (0.16)	N/A^d^	N/A	N/A	N/A
**Spanish, mean (SD)**								
	Validation	0.74 (0.04)	0.79 (0.02)	0.76 (0.03)	.004	.005	.79	.03
	Test	0.62 (0.15)	0.59 (0.16)	0.62 (0.17)	N/A	N/A	N/A	N/A
**Italian & Spanish, mean (SD)**								
	Validation	0.74 (0.02)	0.76 (0.01)	0.74 (0.02)	.06	N/A	N/A	N/A
	Test	0.64 (0.12)	0.65 (0.11)	0.65 (0.13)	N/A	N/A	N/A	N/A

^a^CAT: CatBoost classifier.

^b^SVM: support vector machine.

^c^LR: logistic regression.

^d^N/A: not applicable.

**Table 6:** Classification accuracies on the validation and test sets for the binary classification of group 1 (Mini-Mental State Examination score≥27) versus group 3 (11≤Mini-Mental State Examination score≤19) for the 3 data sets.

Data set		CAT^a^	SVM^b^	LR^c^	*P* value	CAT vs SVM	CAT vs LR	SVM vs LR
**Italian, mean (SD)**								
	Validation	0.92 (0.02)	0.93 (0.02)	0.92 (0.02)	.38	N/A^d^	N/A	N/A
	Test	0.82 (0.14)	0.86 (0.18)	0.89 (0.14)	N/A	N/A	N/A	N/A
**Spanish, mean (SD)**								
	Validation	0.84 (0.03)	0.83 (0.02)	0.82 (0.03)	.17	N/A	N/A	N/A
	Test	0.83 (0.12)	0.73 (0.11)	0.71 (0.15)	N/A	N/A	N/A	N/A
**Italian&Spanish, mean (SD)**								
	Validation	0.85 (0.02)	0.85 (0.01)	0.84 (0.01)	.05	N/A	N/A	N/A
	Test	0.79 (0.11)	0.78 (0.05)	0.81 (0.06)	N/A	N/A	N/A	N/A

^a^CAT: CatBoost classifier.

^b^SVM: support vector machine.

^c^LR: logistic regression.

^d^N/A: not applicable.

## Discussion

An artificial intelligence–based classification pipeline has been implemented to evaluate the possibility of using voice analysis as a prescreening tool for detecting the impairment of cognitive function in a single and multilanguage approach. Multiclass and binary classification were performed on 3 data sets (Italian, Spanish, and a combination of Italian and Spanish data sets). For the multiclass tasks, the models obtained an accuracy of 57%, 53%, and 54% on the test set with SVM on the Italian, Spanish, and multilanguage data set, respectively. Regarding the binary classification, an accuracy of 80%, 59%, and 65% in the test set was achieved on the Italian, Spanish, and multilanguage data set, respectively, when distinguishing between healthy participants and those with the first symptoms of cognitive decline and an accuracy of 86%, 73%, and 78% for the classification between healthy participants and those with an MMSE score≤19. The ROC curves in the multiclass task underlined how the participants with mild symptoms of cognitive decline are the most misclassified. This outcome aligns with expectations since participants belonging to this group exhibit mild impairment, indicated by an intermediate MMSE score. When having a deeper look into the misclassifications results (confusion matrices in Figure 1B and 1D), we observed that for the Italian data set, the model mainly misclassified participants from group 2 with those belonging to group 3, while for the Spanish data set, participants from group 2 were mainly misclassified with participants belonging to group 1. This result is in line with the results of the statistical analysis for the 2 data sets separately, which are reported in Multimedia Appendix 1 (Tables S1-S2): the statistical analysis highlighted a higher number of significantly different acoustic features between group 1 and group 2 for the Italian data set, while for the Spanish data set, there was a higher prevalence of acoustic features, which significantly differed between group 2 and group 3. A possible confounding factor might be the different distributions between the 2 data sets in terms of years of education, with a difference of 5 years between group 1 and group 2 for the Italian data set and a difference of only 1 year for the Spanish data set (Table 2).

The overall differences in the performance between the 2 languages may be explained by the heterogeneous demographic characteristics between the 2 data sets. Indeed, the distribution of participants in terms of gender, which highly affects acoustic features such as pitch [33], differed between the Italian and Spanish data set. In the Italian one, the distribution was more similar among the 3 groups, with a prevalence of females in each group, whereas for the Spanish data set, there was a prevalence of females in group 2 and group 3 compared to group 1, in which there was a balance between the 2 genders. Furthermore, the overall lower performance obtained on the Spanish data set may be related to the distribution of the MMSE scores among the groups. Indeed, there was a sharper separation among the 3 groups in the Italian data set, with a median MMSE score of 30 in group 1 and a median MMSE score of 24 in group 2, whereas the distribution of the scores in the Spanish data set was shrunk, with more participants being borderline among the groups (see Table 2).

The results highlighted that different sets of features are relevant depending on the considered language and the specific task. Indeed, shimmer was shown to be more relevant in Spanish-speaking participants, suggesting that an amplitude variation is predictive of a decline in cognitive function, whereas spectral features and those related to the voiced and unvoiced parts of speech were more important for predicting cognitive decline in Italian-speaking participants. The feature rankings of the classification tasks obtained with the multilanguage data set showed that the most informing features were a combination of those achieved for the 2 languages, when considered individually. This variability in the ranking of the features may be due to the change in prosody and accents of the languages themselves. Indeed, the Italian language is characterized by a wider spectral range compared with Spanish [34], which might explain why spectral features are predominant in the prediction of cognitive decline for the Italian data set. Nevertheless, these speculations need to be further explored in future studies.

Compared to that achieved by Calzà et al [2], we achieved slightly higher performance in the binary classification for distinguishing participants with mild cognitive decline from healthy participants when only the Italian data set was considered. Indeed, we achieved a test accuracy of 80%, while Calzà and colleagues [2] obtained an *F*_1_-score of 75% on a manually checked corpus. However, there are several differences between these 2 studies. First, they considered not only acoustic features extracted from free speech but also lexical and syntactic features extracted with natural language processing as well as the demographic characteristics of the participants, such as age and years of education, which are considered important indicators of cognitive decline [35]. Conversely, we exploited only acoustic features automatically extracted from free speech, without considering any demographic features, to evaluate the possibility of exploiting this method for longitudinal monitoring. Moreover, in their work, Calzà and colleagues [2] recruited participants with a diagnosis of MCI based on a neuropsychological assessment, while in our work, we focused on the prescreening phase before an eventual diagnosis of MCI, and indeed, our groups were discriminated only based on the MMSE score. In another work [24], Bertini et al achieved instead higher performances, that is, 93% after data augmentation with a 20-fold cross-validation with acoustic features extracted from spontaneous speech from a corpus of English-speaking participants, that is, the Pitt Corpus, by applying deep learning techniques on a graphics processing unit. In their study, patients had a diagnosis of AD with the mean MMSE scores of the healthy control group of approximately 29 versus the AD group characterized by a mean score of 18. The lower performances of our model (accuracy of 86% to discriminate between group 1 and group 3 in the Italian data set) may be due to the use of the MMSE score only to distinguish between groups, which may have resulted in misclassification problems. Toth et al [23] achieved 75% accuracy from a binary classification task on Hungarian-speaking participants to distinguish healthy controls from those with MCI by using leave-one-out cross-validation on a set of 88 participants. Our results slightly outperformed their results on the Italian data set (accuracy of 80%), while we achieved lower performances on the Spanish and the multilanguage data sets (accuracy of 62% and 65%, respectively). However, as in the previous studies [2,24], but differently from our study, Toth and colleagues [23] recruited participants with a diagnosis of MCI based on a neuropsychological assessment. Martínez-Sánchez et al [21] classified dementia among Spanish-speaking participants with an accuracy of 80%. The analysis was conducted to distinguish between 35 patients with AD and 35 healthy participants. They stated that fluency is an important aspect of cognitive decline from spontaneous speech, which was confirmed in our work by Figure 1D since the duration of syllables, phonation percentage, and articulation rate are in the top 5 most important features for the multiclass classification of the Spanish data set.

Our approach is based on acoustic features that can be automatically extracted on-the-fly on short speech segments. The satisfactory accuracy achieved with this approach to distinguish healthy participants from those with mild impairment (80% for the Italian data set) makes our results promising toward the design of a mobile app. Leveraging on this tool, an ecological and transparent mass screening of the early signs of cognitive decline can be performed, for example by analyzing free speech during phone calls. Moreover, since there is no need to store raw data and the information content of the speech is not exploited, this tool would preserve the speaker’s privacy.

This work has some limitations. The performance of the models on unseen data, that is, on the test set, worsened overall, probably due to the lack of the generalization power of the model; therefore, there is the need for larger data sets to have more robust classification models. Furthermore, regarding the Spanish data set, another limitation was the lower number of years of education of the recruited participants. Previous studies recruited only participants with more than 6 years of primary education to ensure that participants were fully literate [27]. Another limitation was the use of the MMSE score as the only method to allocate participants into different groups, without collecting information about an eventual diagnosis of MCI or AD, which might have brought to misclassification issues. Furthermore, as reported by Yancheva et al [36], MMSE is affected by a within-participant interrater standard deviation of 3.9 [37,38], which may have resulted in a further wrong group assignment for some participants. Finally, the MMSE test was administered by 2 different professional roles in the 2 recruitment sites—a geriatrician in Italy and a neuropsychologist in Spain, which may have introduced further differences between the 2 data sets.

This work confirmed that it is possible to detect early symptoms of cognitive function decline from the automatic analysis of acoustic features, exploiting a multilanguage approach. Overall, good performances by considering only acoustic features to discriminate between participants with different MMSE scores were achieved. The results obtained on the classification tasks are promising for the development of a screening tool for large-scale monitoring of cognitive function in community-dwelling older adults.

## Appendix

Multimedia Appendix 1 Supplementary results for the binary classification tasks of the Italian and Spanish data sets.

## Abbreviations

AD: Alzheimer disease
CAT: CatBoost classifier
IRCCS: Scientific Institute for Research, Hospitalization and Healthcare
LR: logistic regression
MCI: mild cognitive impairment
MMSE: Mini-Mental State Examination
ROC: receiver operating characteristic
SHAP: Shapley Additive Explanations
SVM: support vector machine
