# Supplementary material for: Automatic Spontaneous Speech Analysis for the Detection of Cognitive Functional Decline in Older Adults: Multilanguage Cross-Sectional Study
Source: JMIR Aging. 2024 Apr 29;7:e50537. doi: 10.2196/50537 (PMC11091799; doi:10.2196/50537)
Supplement: Multimedia Appendix 1 [file aging_v7i1e50537_app1.pdf]

Supplementary Materials

Acoustic feature characteristics for the Italian and Spanish datasets are reported in Tables S1 and S2, respectively. Table S1 highlights how features from all the acoustic domains apart from shimmer contribute to the distinction between Group 1, made up of healthy subjects, i.e., MMSE≥27 and Group 2 (subjects with mild impairment, 20≤MMSE≤26). Indeed, unvoiced features resulted in being highly significant along with the syllabic (duration of syllables and pauses) and spectral features. Similar trends can be found between Group 1 and Group 3 (subjects with severe impairment, 10≤MMSE≤19). In contrast, acoustic features are found to be more similar between Group 2 and Group 3.

In Table S2 for the Spanish dataset, unlike the Italian set, the features between Group 1 and Group 2 are more similar overall, with a higher discriminative power between Group 2 and Group 3. Nevertheless, the mean duration between syllables and duration of pauses were significant in distinguishing Group 1 from 2, as well as the spectral centroid and standard deviation of the 3rd formant.

Figure S1 displays the ROC curves and confusion matrices of the binary classification task distinguishing Group 1 from Group 2 for the Italian and Spanish datasets obtained with Support Vector Machine. The ROC curve in Figure S1a shows a fair AUC for the Italian subjects, while there is a worsening of performance in Figure S1c for the Spanish dataset. These results are confirmed by the confusion matrix in S1b which reports a lower number of misclassified subjects for the Italian dataset, compared to the matrix in Figure S1d of the Spanish dataset.

The feature rankings in Figure S2a and Figure S2b confirm that different types of features are affected by cognitive decline depending on the language. Indeed, the most affected features, according to the SHAP method, are those related to the spectrum among the Italian subjects, whereas shimmer-related features and voice periodicity among the Spanish participants.

An overall improvement concerning the ROC curves is shown in Figure S3 (a and c), reaching an AUC of 0.86 and 0.70 in the classification among participants of Group 1 and those from Group 3, respectively for the Italian and Spanish datasets. These results are also supported by the confusion matrices in Figure S3b for the Italian dataset in which only 12 subjects were misclassified and Figure S3d for the Spanish dataset in which 24 out of 88 subjects were confused.

As for the multiclass classification and the previous binary classification task, the feature rankings in Figure S4a and Figure S4b, respectively for the Italian and Spanish datasets, confirm the different importance of the features for predicting cognitive decline depending on the language. Indeed, the most affected features are syllabic and voice periodicity among the Italian subjects (Figure S4a), whereas shimmer-related and syllabic features among the Spanish participants (Figure S4b), corroborating the statistical analysis results in Table S2.

Table S1. Acoustic features characteristics and significance among the three groups for the Italian dataset.

Domain	Feature*	Group 1 (n=45)	Group 2 (n=44)	Group 3 (n=44)	P-value	P-value G1vsG2	P-value G2vsG3	P-value G1vsG3
Voice Periodicity								
	Unvoiced [%]	32.4 (10.6)	43.7 (15.3)	48 (11)	<.001	<.001	.50	<.001
	Duration of voiced							

	segments [s]								
		Mean	1.04 (0.38)	0.9 (0.43)	0.83 (0.27)	.018	.21	.99	.02
		Median	0.83 (0.34)	0.71 (0.34)	0.66 (0.22)	.021	.15	1	.02
		15th percentile	0.24 (0.08)	0.22 (0.11)	0.2 (0.06)	.055			
		85th percentile	1.98 (0.77)	1.71 (0.86)	1.57 (0.55)	.027	.29	.98	.03
	Duration of unvoiced segments [s]								
		Mean	0.46 (0.12)	0.67 (0.23)	0.78 (0.22)	<.001	<.001	.04	<.001
		Median	0.32 (0.08)	0.44 (0.16)	0.49 (0.17)	<.001	<.001	.30	<.001
		15th percentile	0.14 (0.01)	0.16 (0.04)	0.16 (0.02)	<.001	<.001	1	<.001
		85th percentile	0.86 (0.27)	1.32 (0.54)	1.59 (0.54)	<.001	<.001	.03	<.001
	Voice Breaks [%]		33.9 (10.3)	45 (14.7)	49.2 (10.8)	<.001	<.001	.47	<.001
**Shimmer**	Shimmer [dB]		5.04 (0.55)	5.08 (0.79)	5.2 (0.54)	.49			
**Syllabic and Pauses features**									
	Speech Rate [syl/s]		4.23 (0.54)	3.68 (0.7)	4.66 (9.51)	.06			
	Phonation [%]		74.6 (6.7)	65 (9.9)	74.9 (99.7)	.10			
	Articulation Rate [syl/s]		5.68 (0.42)	5.68 (0.57)	5.42 (0.44)	.01	1	.03	.03
	Mean duration between syllables [s]		0.69 (0.18)	0.94 (0.32)	1.1 (0.3)	<.001	<.001	.02	<.001
	Mean duration of syllables [s]		0.14 (0.01)	0.15 (0.02)	0.16 (0.02)	<.001	.008	.02	<.001
	Number of pauses		4.49 (1.34)	4.57 (1.58)	5.25 (1.25)	.06			
	Mean duration of pauses [s]		0.53 (0.17)	0.71 (0.3)	0.87 (0.29)	<.001	<.001	.02	<.001
**Spectral features**									
	Pitch [Hz]								
		Mean	160.9(17.8)	161.7(25.9)	161.3(18.3)	.98			
		Standard deviation	65.3 (8.7)	71.6 (12.7)	79.5(13.5)	<.001	.02	.007	<.001
	SD-F3 [Hz]		459 (41)	481 (44)	490 (44)	.002	.46	1	.002
	Speech temporal regularity		1765 (62)	1718 (69)	1710 (65)	<.001	.002	1	<.001
	Spectral centroid [Hz]		783 (147)	693 (135)	665(106)	<.001	.004	.84	<.001

*mean (SD)

Table S2. Acoustic feature characteristics and significance between the three groups for the Spanish dataset.

Domain	Feature*		Group 1 (N=43)	Group 2 (N=45)	Group 3 (N=45)	P-value	P-value G1vsG2	P-value G2vsG3	P-value G1vsG3
**Voice periodicity**									
	Unvoiced [%]		33.1 (10.1)	32.8 (8.7)	41.5 (11.6)	<.001	1	<.001	.001
	Duration of voiced segments [s]								
		Mean	1.13 (0.34)	1.15 (0.33)	1.12 (1.06)	.94			
		Median	0.93 (0.29)	0.93 (0.33)	0.93 (1.02)	1			
		15th percentile	0.29 (0.11)	0.26 (0.1)	0.23 (0.14)	.04	.63	.54	.03
		85th percentile	2.07 (0.63)	2.14 (0.57)	1.97 (1.38)	.563			
	Duration of unvoiced segments [s]								
		Mean	0.58 (0.16)	0.58 (0.15)	0.73 (0.27)	<.001	1	.001	.001
		Median	0.47 (0.15)	0.46 (0.12)	0.55 (0.2)	.008	1	.01	.04
		15th percentile	0.16 (0.03)	0.16 (0.02)	0.17 (0.04)	.08			
		85th percentile	1.07 (0.31)	1.09 (0.31)	1.42 (0.62)	<.001	1	<.001	<.001
	Voice Breaks [%]		34.5 (9.5)	34.9 (8.5)	45.2 (10.6)	<.001	1	<.001	<.001
**Shimmer**	Shimmer [dB]		4.96 (0.56)	5.3 (0.52)	4.89 (0.88)	<.001	.09	.03	1
**Syllabic and pauses features**	Speech Rate [syl/s]		3.59 (0.43)	3.35 (0.5)	2.92 (0.67)	<.001	.19	.001	<.001
	Phonation [%]		65.26 (6.08)	62.37 (8.1)	54.02 (9.44)	<.001	.40	<.001	<.001
	Articulation Rate [syl/s]		5.54 (0.44)	5.39 (0.36)	5.41 (0.49)	.22			
	Mean duration between syllables[s]		0.85 (0.18)	1.01 (0.28)	1.38 (0.57)	<.001	.03	<.001	<.001
	Mean duration of syllables [s]		0.15 (0.01)	0.15 (0.01)	0.16 (0.02)	<.001	.08	.13	<.001
	Number of pauses		5.71 (0.78)	5.62 (1.02)	5.39 (1.2)	.35			
	Mean duration of pauses [s]		0.63 (0.14)	0.77 (0.28)	0.96 (0.33)	<.001	.01	.002	<.001
**Spectral features**	Pitch [Hz]								
		Mean	162.8 (29.9)	170.3(26.2)	154.6 (29.9)	.04	.73	.03	.54
		Standard deviation	71.8 (14)	73.2 (11.9)	78.4 (17.2)	.073			
	SD-F3 [Hz]		472 (50)	498 (47)	480 (49)	.04	.04	.23	1
	Speech temporal regularity		1733(66)	1715 (64)	1666 (96)	<.001	.83	.009	<.001
	Spectral centroid [Hz]		834 (157)	858 (150)	845 (216)	.81	.004	.84	<.001

*mean(SD)

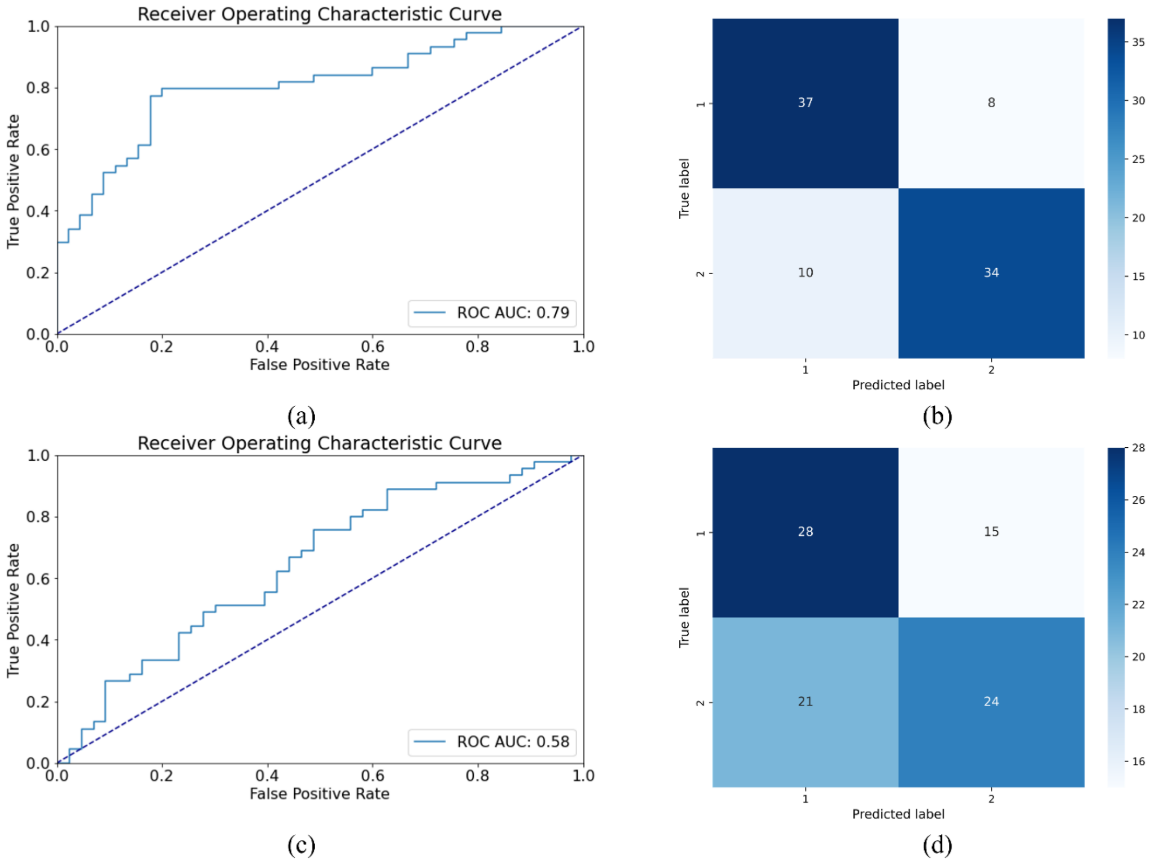

Figure S1. ROC curves (a, c) and confusion matrices (b, d) for binary classification between Group 1 (MMSE≥27) and Group 2 MMSE (20≤MMSE≤26) of the Italian (a, b) and Spanish (c, d) datasets.

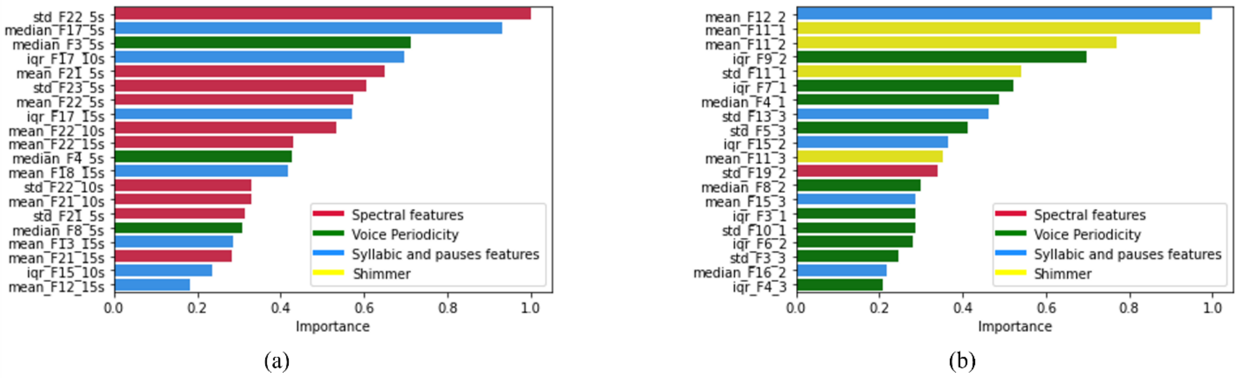

Figure S2. Feature ranking of binary classification between Group 1 and Group 2 for Italian (a) and Spanish (b) datasets. The ranking is represented from the most to the least important feature with colors corresponding to the domains reported in the legend.

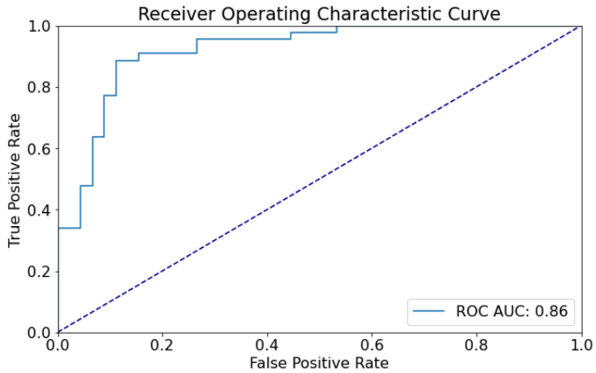

(a)

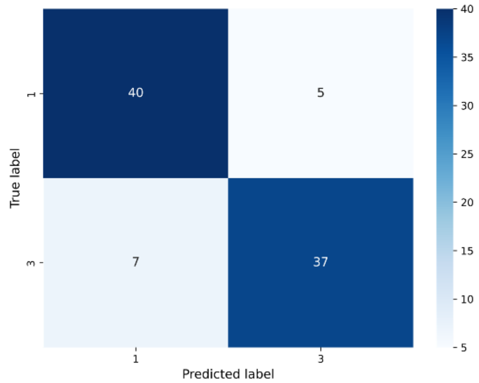

(b)

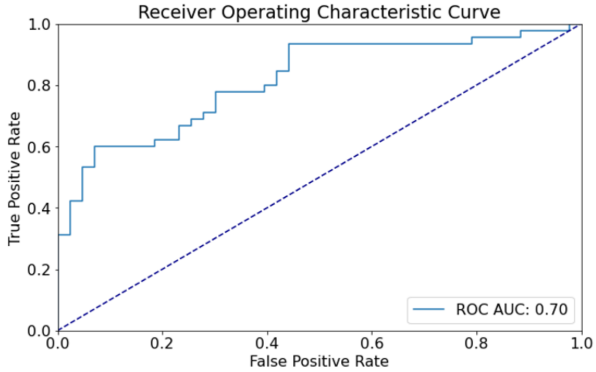

(c)

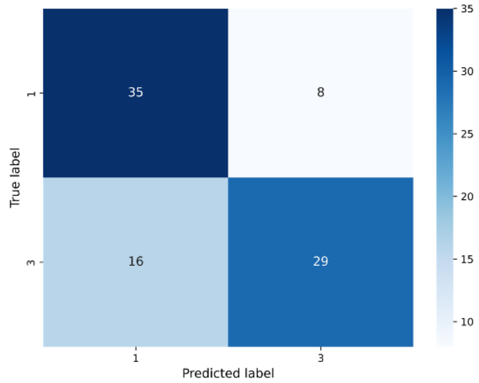

(d)

Figure S3. ROC curves (a, c) and confusion matrices (b, d) for binary classification between Group 1 (MMSE≥27) and Group 3 MMSE (MMSE≤19) of the Italian (a, b) and Spanish (c, d) datasets.

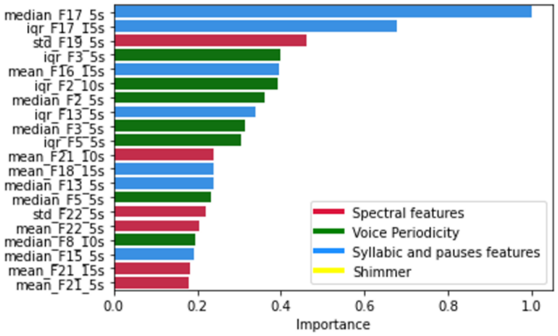

(a)

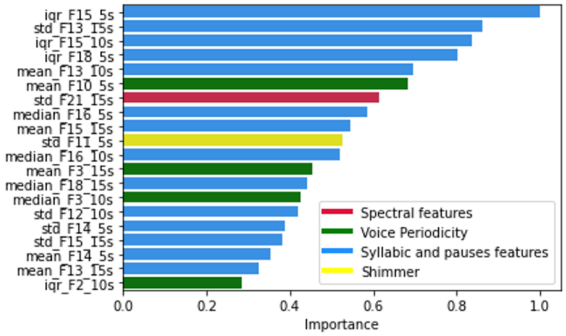

(b)

Figure S4. Feature ranking of binary classification between Group 1 and Group 3 for Italian (a) and Spanish (b) datasets. The ranking is represented from the most to the least important feature with colors corresponding to the domains reported in the legend.
